# One-step zero-background IgG reformatting of phage-displayed antibody fragments enabling rapid and high-throughput lead identification

**DOI:** 10.1093/nar/gkt1142

**Published:** 2013-11-16

**Authors:** Chao-Guang Chen, Louis J. Fabri, Michael J. Wilson, Con Panousis

**Affiliations:** Research and Development, CSL Limited, 30 Flemington Road, Parkville, Victoria 3010, Australia

## Abstract

We describe a novel cloning method, referred to as insert-tagged (InTag) positive selection, for the rapid one-step reformatting of phage-displayed antibody fragments to full-length immunoglobulin Gs (IgGs). InTag positive selection enables recombinant clones of interest to be directly selected without cloning background, bypassing the laborious process of plating out cultures and colony screening and enabling the cloning procedure to be automated and performed in a high-throughput format. This removes a significant bottleneck in the functional screening of phage-derived antibody candidates and enables a large number of clones to be directly reformatted into IgG without the intermediate step of *Escherichia coli* expression and testing of soluble antibody fragments. The use of InTag positive selection with the Dyax Fab-on-phage antibody library is demonstrated, and optimized methods for the small-scale transient expression of IgGs at high levels are described. InTag positive selection cloning has the potential for wide application in high-throughput DNA cloning involving multiple inserts, markedly improving the speed and quality of selections from protein libraries.

## INTRODUCTION

Since the initial finding that filamentous phages were capable of expressing heterologous peptides on their surface (1) and that functional antibody fragments could be assembled in *Escherichia coli* (2,3) and expressed on the surface of fd bacteriophage (4), phage display of antibody fragments has evolved as an important tool in the discovery of human therapeutic antibodies. Over the past three decades, a number of methods have been used to generate large Fab or scFv-based phage display libraries of human antibodies, which attempt to mimic the sequence and structural diversity of the human immunological repertoire (5). These include libraries constructed using variable region genes fully derived from human donors (6), semi-synthetic libraries where diversity is attained through a combination of synthetic and donor-derived variable region components (7) and fully synthetic libraries where germline usage and amino acid composition of complementarity-determining regions are either randomized (8) or rationally based on naturally occurring amino acid sequences in the human population (9).

Screening of antibody phage display libraries for clones with specificity to a target antigen involves iterative rounds of antigen binding and phage amplification. The use of high-throughput (HTP) screening technologies enables thousands of phage clones to be readily screened for antibodies with specificity to a target antigen (10–12). However, the functional evaluation of antibodies while still fused to the bacteriophage is limited and generally requires the re-engineering of phage clones to enable expression and purification of soluble recombinant antibody fragments for analysis, typically in *E. coli*. This process is time-consuming and can be problematic in regard to yield, particularly for mammalian-derived antibody libraries where codon usage favours mammalian expression. Furthermore, lipopolysaccharide contamination from bacterial production can interfere with *in vitro* cellular assays and *in vivo* functional screening. For comprehensive antibody characterization, particularly where the final therapeutic format is whole immunoglobulin G (IgG), it is preferable that the antibodies are reformatted directly into IgG molecules and expressed in mammalian cells. This is particularly relevant for assessing functional activities requiring the antibody Fc region such as immunological effector functions, but also where avidity is required for biological function, e.g. receptor cross-linking. However, owing to the lack of rapid and HTP IgG reformatting methods, the *E. coli* expression step is currently required to narrow the number of lead candidates before IgG reformatting and mammalian expression.

The HTP reformatting of antibody fragments for expression in an IgG format presents some significant challenges. In regard to cloning, two genes (encoding the light chain and heavy chain) need to be cloned for the expression of each antibody. Furthermore, the cloning requires the perfect in-frame fusion of the variable antibody regions from the phage display vector with the light and heavy chain IgG constant regions and signal peptides in the mammalian expression vector. Commonly used IgG reformatting methods have been reported where the heavy and light chain immunoglobulin genes are generated in separate vectors and IgG expressed following co-transfection in mammalian cells (13), or sequentially cloned into a single mammalian dual-expression vector (14–16). A single dual-expression vector is preferable to two separate vectors in an HTP process, as it decreases the number of vectors that need to be generated and improves the process speed and reagent requirements. Importantly, it also minimizes potential errors in maintaining the original phage-derived antibody heavy and light chain pairings throughout vector construction and protein expression.

The key limitations for both of these cloning strategies are the use of restriction digestion for the preparation of variable region inserts from the phage-display vectors, which can result in the loss of clones containing internal restriction sites; the high cloning background, which results from uncut and re-ligated vector; and the multiple cloning steps required. A ligation-independent cloning (LIC) method has been reported for antibody reformatting (17), which overcomes the potential loss of clones containing internal restriction sites but does not address high cloning background and relies on separate expression vectors for the light and heavy antibody chains. Hence, alternative cloning strategies are required to support HTP requirements.

To overcome these problems, we have developed an insert-tagged (InTag) positive selection method where a positive selection marker (e.g. chloramphenicol-resistance gene) is cloned together with the other inserts required for IgG reformatting into a single mammalian expression vector. This enables recombinant clones to be selected without cloning background. InTag positive selection bypasses the need to plate out cultures and screen colonies, thus allowing the cloning procedure to be automated and performed in an HTP format. This method is reliable and facilitates the expression of IgG reformatted antibodies at levels sufficient for extensive functional evaluation from a single batch (generally >0.5 mg), improving the speed and quality of selections from protein libraries.

## MATERIALS AND METHODS

### Preparation of CmR InTag adaptor

Refer to Supplementary Figure S1 for a schematic representation of the InTag adaptor and to Supplementary Table S2 for primer sequences.

1. Amplify the BGH pA fragment from pcDNA3.1 (Invitrogen) by polymerase chain reaction (PCR) using primers 1 and 2 (Supplementary Table S2).
2. Amplify the CmR gene from the Gateway vector conversion system (Invitrogen) using primers 3 and 4.
3. Amplify the CMV promoter region from pcDNA3.1 (Invitrogen) using primers 5 and 6.
4. Amplify the signal peptide from the DYAX IgG4 vector (16) using 7 and 8 primers.
5. Perform the PCR in 50-μl PCR reaction containing 10 ng of template DNA, 1× AccuPrime Mix (Invitrogen), 0.5 μM of each primer and 1 U of pfx DNA polymerase (Invitrogen). PCR cycling conditions are as follows: 94°C for 30 s; 5 cycles of 93°C for 30 s, 50°C for 30 s and 68°C for 1 min; 25 cycles of 93°C for 20 s; 60°C for 30 s and 68°C for 1 min; and 68°C for 10 min.
6. Join the four DNA fragments by SOE-PCR in a step-wise manner. Firstly join PCR products from steps (1) and (2) using primers 1 and 4, and steps (3) and (4) using primers 5 and 8 respectively, before combining to form the final adaptor with primers 1 and 8. Perform the PCRs as in step 5 using 2 µl each of the relevant PCR products as DNA template.
7. Subclone the fragment into pCR4Blunt-TOPO vector (Invitrogen) according to the manufacturer’s instruction for sequence analysis.

### Preparation of ZeoR InTag adaptor

Refer to Supplementary Figure S1 for a schematic representation of the InTag adaptor and to Supplementary Table S2 for primer sequences.

1. Amplify the BGH pA from pcDNA3.1 by PCR using primers 1 and 9.
2. Amplify the ZeoR marker gene from pCR-BluntII-TOPO (Invitrogen) using primers 10 and 11.
3. Amplify the CMV promoter region from pcDNA3.1 using primers 12 and 6.
4. Amplify the signal peptide from the DYAX IgG4 vector (16) using 7 and 8 primers.
5. Join the four DNA fragments by SOE-PCR and then subclone into pCR4Blunt-TOPO vector as above.

### IgG reformatting

1. Digest 5 μg of vector with ApaLI and NheI and 5 ug of InTag adaptor plasmid with AscI and MfeI. Gel-purify the vector and isolate the DNA using QIAquick gel extraction kit (QIAGEN). Quantitate the vector and the InTag adaptor.
2. Dilute the overnight phage culture with LB media by 1/25 and use 2 µl for PCR.
3. Amplify the light chain with primers P1 and P2 (Supplementary Table S1), and amplify the VH using primers P3 and P4 (Supplementary Table S1) in a single tube.
4. PCR was carried in a 20-μl PCR reaction containing 1× AccuPrime Mix (Invitrogen), 0.5 μM of each primer (P1, P2, P3 and P4; Supplementary Table S1) and 1U of pfx DNA polymerase (Invitrogen). PCR cycling are as following: 94°C for 30 s; 5 cycles of 93°C for 30 s, 50°C for 30 s and 68°C for 1 min; 25 cycles of 93°C for 20 s; 60°C for 30 s and 68°C for 1 min; and 68°C for 10 min.
5. Treat 5 µl of PCR products with 2 µl of Cloning Enhancer (Clonetech) at 37°C for 15 min and 80°C for 15 min.
6. Set up a 10-μl In-Fusion reaction using 1 µl enhancer-treated PCR product, 50 ng vector, 50 ng InTag adaptor, 1× In-Fusion HD enzyme premix and water. Incubate in 50°C for 15 min. Leave on ice or store at −20°C.
7. Add 2 μl of the In-Fusion reaction mixture into 20 μl of DH5a chemical competent cells (Bioline) or Top10 (Invitrogen), incubate on ice for 30 min, heat shock at 42°C for 45 s, return to ice for 2 min, add 80 μl of SOC and recover at 37°C for 1 h without shaking.
8. Transfer the 100-μl cells into 5 ml of LB containing 34 ng/ml chloramphenicol; incubate in 37°C for overnight to 2 days*.*
9. Isolate Miniprep DNA from 2-ml liquid cultures using the QIAprep Spin Miniprep kit (QIAGEN) for sequencing analysis and transient transfection.

### Transient transfection

1. Culture FS 293 cells (Invitrogen) in FS 293 Expression media (Invitrogen) supplemented with 10 ml/L antibiotic/antimycotic solution to log phase.
2. Centrifuge cells at 1200 rpm for 5 min and resuspend in correct volume of FS 293 Expression media.
3. Dilute 10 μg plasmid DNA in 1 ml Opti-MEM (Invitrogen), mix gently and incubate for 5 min at room temperature.
4. Dilute 20 μL 293fectin (Invitrogen) in 1 ml Opti-MEM, mix gently and incubate for 5 min at room temperature.
5. Add the diluted DNA to the diluted 293fectin, mix gently and incubate for 10–60 min at room temperature to allow DNA-293fectin complexes to form.
6. Add the DNA-293fectin complex to a flask containing 3 × 10^7^ cells in 28 ml of FS 293 Expression media supplemented with 10 ml/L antibiotic/antimycotic solution. Incubate the cells at 37°C with shaking.
7. Add 750 μl LucraTone™ Lupin (Celliance) to the 30 ml culture 4–24 h post-transfection.
8. Collect the supernatants and filter through 0.45 polyvinylidene difluoride low protein-binding filter before quantitation and purification.

### Protein purification

Monoclonal antibodies are purified using an AKTA express (GE Healthcare, UK) as per the manufacturers’ recommended method.

1. HiTrap MabSelect SuRe (1 ml, GE Healthcare, UK) columns equilibrated with MT-phosphate buffered saline (PBS) buffer.
2. Filtered conditioned cell culture media is loaded on to the column at a flow rate of 1 ml/min and washed sequentially with 10 column volumes (CV) of MT-PBS and 6 CV of 10 mM Tris, 0.5 M arginine, 150 mM NaCl, pH 7.2.
3. Bound antibody is eluted with 5 CV of 0.1 M Na acetate, pH 3.0, and the peak fraction immediately applied to a HiPrep 26/10 desalting column (GE Healthcare, UK) pre-equilibrated with MT-PBS.
4. Purified antibody is quantified by absorbance at 280 nm using a DropSense96 (Trinean) spectrophotometer.
5. Protein fractions are pooled and concentrated using an Amicon Ultracel 50 K centrifugal device (Millipore) before sterile filtration with 0.22-µm filters. The purified antibody is analysed by sodium dodecyl sulphate-polyacrylamide gel electrophoresis (reducing and non-reducing) and analytical size exclusion chromatography for the presence of high- and low-order aggregates, respectively.

### Light chain swapping (Supplementary Figure S2)

1. Remove the original light chain (VL1/CL1) and CmR InTag adaptor by digestion with ApaLI and MfeI. Gel-purify the vector background.
2. Isolate the new light chain (VL2/CL2) by ApaLI and AscI digestion.
3. Isolate the ZeoR InTag adaptor by AscI and MfeI digestion.
4. Ligate the new light chain and ZeoR InTag adaptor with the plasmid digested with ApaLI and MfeI using T4 DNA ligase (Promega).
5. Select the recombinant plasmid with Zeocin (Invitrogen).

## RESULTS

### Zero-background IgG reformatting using InTag positive selection

**Figure 1.**
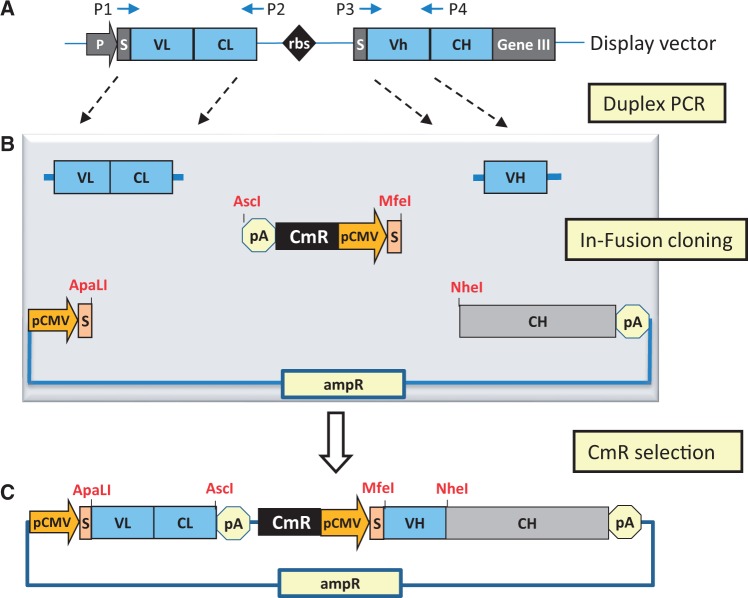
Schematic representation of zero-background IgG reformatting using InTag positive selection. (**A**) Amplification of variable antibody regions from phagemid. The light chain and VH-encoding regions are amplified from the phage display construct by duplex-PCR with primers P1, P2, P3 and P4 (Supplementary Table S1). (**B**) In-Fusion cloning of variable antibody regions using InTag positive selection. PCR products from (A) were treated with cloning enhancer and mixed with pre-prepared InTag adaptor, mammalian expression vector (with heavy chain constant region) and the In-Fusion cloning enzyme. The resulting DNA was transformed into *E. coli*, and recombinant plasmids were selected in liquid media containing chloramphenicol. P: promoter; S: signal peptide; rbs: ribosome binding site; pA: polyadenylation signal, pCMV: CMV promoter, CmR: chloramphenicol-resistance marker, AmpR: ampicillin resistance marker. (**C**) Final IgG reformatted mammalian expression vector.

A general scheme for the InTag-based IgG reformatting of ‘Fab-on-phage’ clones from the Dyax fully human antibody library (7) is shown in Figure 1 (see ‘Methods’ section for detailed protocols). The initial step (Figure 1A) involves amplification of the antibody light chain and the variable heavy region by duplex-PCR from the phagemid vector using the specified primers (Supplementary Table S1). These primers have been adapted from (16) to facilitate ligation-independent In-Fusion™ (Clontech) cloning, using 15-bp extensions complementary to the mammalian expression vector and InTag adaptor. Without need for purification and restriction digestion, the duplex-PCR products are treated with cloning enhancer before being directly added to InTag adaptor and ApaLI/NheI linearized mammalian expression vector for In-Fusion cloning (Figure 1B). Chemically competent bacteria are transformed with the In-Fusion reaction mixture and then placed directly into LB liquid cultures containing chloramphenicol. The design of the InTag adaptor and cloning strategy ensures that only clones with all three inserts correctly orientated will survive under chloramphenicol selection and hence the functionally assembled IgG expression vector (Figure 1C) can be prepared from the liquid cultures for use in mammalian expression without the need for plating-out and screening of bacterial colonies for correct clones.

The InTag adaptor serves two key roles in this one-step cloning strategy: first, it provides the necessary regulatory elements (polyadenylation site for the light chain and the CMV promoter and mammalian signal peptide for the antibody heavy chain) to facilitate the high-level expression of IgG in mammalian cells; second, it serves as an antibiotic resistance marker to facilitate the positive selection of reformatted clones. A schematic representation for the InTag adaptor based on chloramphenicol or zeocin selection markers is shown in Supplementary Figure S1. Primers used in the construction of InTag adaptor are listed in Supplementary Table S2 and protocols for its preparation are detailed in the Methods section. We routinely use the chloramphenicol InTag adaptor for IgG reformatting of ‘Fab-on-phage’ clones, although InTag adaptors with a different selection marker (e.g. zeocin) can also be used. We also use InTag positive selection cloning for further optimization of lead IgG antibody candidates using heavy or light chain shuffling methodologies (18). The alternate use of InTag adaptors with different selection markers enables the chain shuffling to be undertaken sequentially, rapidly and with high fidelity, given the absence of cloning background from the parental IgG expression vector containing the chloramphenicol selection marker (data not shown). An example of the use of the InTag cloning for antibody light chain shuffling is shown in schematic form in Supplementary Figure S2.

The primers used in the duplex PCR amplification of light chain and variable heavy chain regions are tailored for use with the DYAX ‘Fab-on-phage’ antibody library. Given the diversity of construction methods and antibody variable regions used in the generation of other antibody phage libraries, primers listed in Supplementary Table S1 need to be modified accordingly. For Fab-based phage libraries, additional P1 and P3 primers (non-underlined regions) can be generated to accommodate all human germline sequences used in the library construction. In the case of scFv libraries, the non-underlined region of the P2 primer can be modified to complement the C-terminal region of the VL regions rather than the constant light chain region used for Fab-based libraries. The mammalian expression vector used for the IgG reformatting has been modified to contain the human heavy chain constant regions (CH1-hinge-CH2-CH3) as described previously (16). This expression vector is based on the pCMV/myc/ER vector from Invitrogen™ and is provided with the DYAX library in an IgG1 or IgG4 format. Other mammalian expression vectors can be readily adapted for use with InTag cloning via the insertion of a heavy chain constant region (species and isotype of choice) and subsequent modification of the complementary P1 and P4 primer sequences (double-underlined in Supplementary Table S1). We have modified a pcDNA3.1 mammalian expression vector (Invitrogen™) in this manner with similar results in regard to cloning efficiency and antibody yield (data not shown).

### Comparison of InTag IgG reformatting with traditional cloning methods

**Figure 2.**
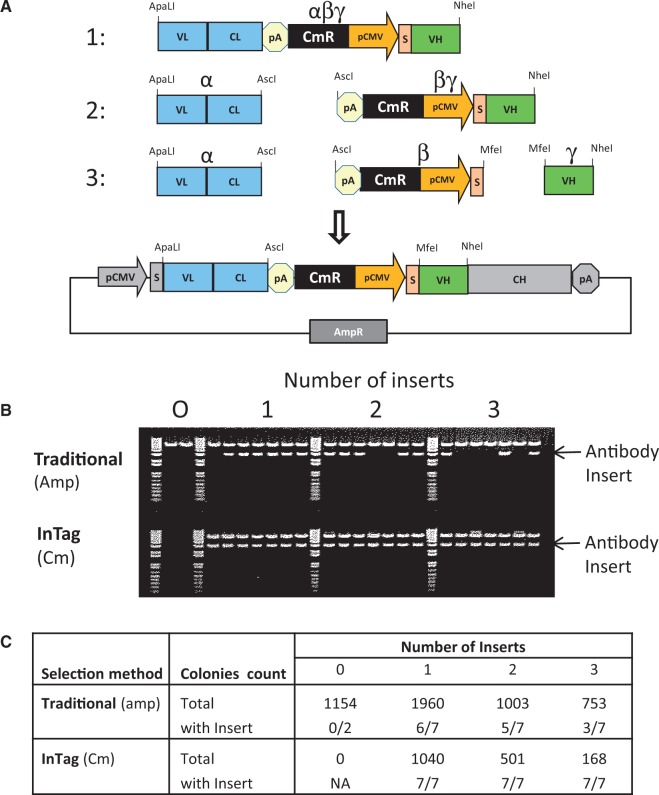
Elimination of cloning background by InTag positive selection. (**A**) Cloning strategy. An antibody cassette containing a CmR selection marker was cloned using T4 DNA ligase into a vector containing the AmpR marker as a purified single fragment (1), two separate fragments (2) or three separate fragments (3). Each ligation contained 50 ng of vector DNA and 60 ng αβγ insert, 20 ng α and 40 ng βγ inserts, or 20 ng α, 30 ng β and 10 ng γ inserts, for each of the three cloning strategies, respectively. The transformed cells were plated onto agar plates containing either chloramphenicol (InTag positive selection) or ampicillin (traditional selection). (**B**) Restriction digestion analysis. The miniprep DNA was digested with ApalI and NheI and run on a 1% agarose gel. (**C**) Summary of results.

Traditional cloning methods rely on a selection marker (e.g. AmpR) located on the vector backbone to select for successfully transformed bacterial clones. Cloning background resulting from uncut or re-ligated parental plasmid is a major disadvantage of this method and necessitates plating-out and screening of individual bacterial colonies for correct clones. Using our InTag method, a positive selection marker (e.g. CmR) is co-inserted into the plasmid during the cloning process and removes background cloning by selecting against uncut or re-ligated parental plasmid. To demonstrate the advantage of the InTag method compared with traditional cloning strategies, the insert encoding the antibody shown in Figure 2A was isolated as a single fragment (αβγ), two separate fragments (α and βγ) or three separate fragments (α, β and γ) and cloned into a mammalian expression vector using T4 DNA ligase. The three ligations were transformed into chemically competent Top10 cells (Invitrogen™) and selected on agar plates containing either chloramphenicol (InTag positive selection) or ampicillin (traditional selection). As a control, cells were also transformed with linearized and gel-purified mammalian expression vector alone. The colonies on each plate were counted, and plasmid DNA was also isolated from seven colonies and subjected to restriction digest analysis (Figure 2B–C). Using traditional ampicillin selection, >1000 colonies were obtained with the digested expression vector alone and none with the InTag positive selection, as expected (Figure 2C). From the ligation plates with 1, 2 and 3 inserts, 6/7, 5/7 and 3/7, respectively, of clones randomly picked from the traditional selection strategy contained insert. As expected using traditional cloning, as the number of inserts to be cloned increased so did the cloning background. In comparison, 100% of all the clones randomly selected from the three InTag positive selection plates were shown to contain the correct insert following restriction digestion (Figure 2B–C), indicating that cloning background from uncut or re-ligated vector has been eliminated using the InTag method.

**Figure 3.**
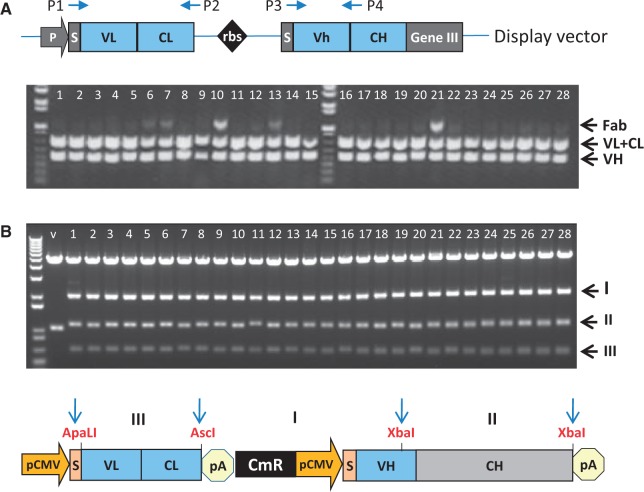
HTP IgG reformatting of 28 phagemid clones using LIC and InTag positive selection. (**A**) Duplex PCR. PCR products for the light chain (VL + LC, primers P1 and P2) and VH (primers P3 and P4) amplifications are shown. Because starting phage levels are not normalized, a larger fragment corresponding to the Fab (product of P1 and P4 primers) is present in some samples. This fragment can be cloned in the vector but will be eliminated by chloramphenicol selection as it does not contain the selection marker. (**B**) Restriction digestion analysis. Plasmid DNA from all 28 clones was digested with ApaLI, AscI and XbaI and resolved by agarose gel electrophoresis. Expected fragment profiles (I–III) for successfully reformatted clones are shown in the schematic representation below the gel. For vector (V) only control, an ApaLI and XbaI fragment consisting mainly of the CH was produced as expected. M: 1 kb plus DNA markers (Invitrogen™); V: vector.

Having shown the benefits of InTag over traditional cloning, we selected 28 unique phage clones from the DYAX library and converted these to an IgG format with the HTP InTag method using LIC, rather than the traditional ligation exemplified in Figure 2. As shown in Figure 3, restriction digest analysis of plasmid DNA from the 28 clones shows that all were successfully reformatted, supporting the use of LIC together with InTag cloning for the HTP IgG reformatting of phage-displayed antibody fragments.

To date, we have reformatted >2000 clones using inTag positive selection and have found the method to be highly robust and reproducible. However, at times the phagemid templates from the phage library screening step lack clonality, resulting in the detection of multiple sequences following InTag cloning. These clones can either be omitted from further characterization or plated out to produce single colonies. Because InTag positive selection eliminates the cloning background, only a small number of colonies need to be sequenced to identify the correct clone. When initially implementing InTag-based cloning strategies into other laboratories, we recommend PCR conditions are optimized and the highest quality PCR primers and template DNA are used to ensure fidelity.

### Optimization of transient IgG expression

Having developed an HTP IgG reformatting strategy, we sought to optimize an automation-compatible process for the mammalian expression of IgG candidates (based on the Freestyle™ 293 expression system from Invitrogen™) at levels sufficient (>0.5 mg) to facilitate extensive functional and biophysical characterization from a single 30-ml culture (see ‘Methods’ section for detailed protocols). This amount of material enables all antibody candidates to undergo accurate determination of binding affinity and extensive functional screening using a panel of cell-based biological potency assays, with adequate replicates and concentrations to facilitate the robust selection of lead candidates for further analysis.

#### Lupin addition

**Figure 4.**
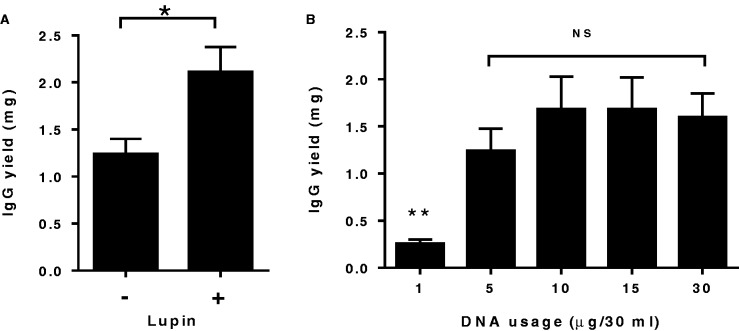
Optimization of transient IgG expression levels in 293FS cells. Seven independent phage clones following InTag IgG reformatting were transiently transfected in 30-ml cultures under various conditions and their total IgG expression levels were compared. Data show the mean ± SEM IgG yields for the seven clones. (**A**) Effect of lupin addition. The addition of lupin has significantly increased the antibody yields by an average of 70%. *P < 0.05, Student’s unpaired *t*-test. (**B**) DNA usage. With the exception of 1 µg DNA, there was no significant difference in the IgG expression levels using the 5, 10, 15 µg when compared with that obtained using 30 µg per 30 ml of DNA, **P < 0.01, one-way analysis of variance with Dunnett’s multiple comparisons test. NS: no significance.

The effect of *Lucra*Tone™ lupin plant hydrosylates on IgG yields was examined, as the addition of protein hydrosylates has been reported to significantly increase transient recombinant protein expression in HEK293 cells (19). Following the addition of 0.5% w/v of lupin 2–4 h after transient transfection, expression yields were improved by an average of 70% (Figure 4A).

#### DNA usage

Invitrogen™ recommends the use of 20–40 µg of DNA (30 µg typically) with their Freestyle™ 293 expression system in 30-ml cultures. However, given a typical plasmid miniprep only produces up to 20 µg of plasmid, we often needed to perform two or more mini-preparations to carry out a 30-ml transfection. To overcome this significant bottleneck for HTP application, we examined DNA usage in the transfection protocol. Figure 4B shows the average expression results for seven independent IgG constructs using 1, 5, 10, 20 and 30 µg of each expression vector in 30 ml cultures. From this experiment, it can be seen that the use of 5, 10 or 15 µg of DNA gave equivalent IgG expression levels to the recommended 30 µg. Because 10 µg of plasmid DNA is readily obtained from a single plasmid mini-preparation, we selected this level for use in our optimized transfection protocol.

#### 293fectin™ usage and DNA/293fectin™ incubation time

293fectin™ reagent contributes significantly to the overall cost associated with transient transfection. Because our HTP IgG reformatting method can readily generate hundreds of antibody candidates for mammalian expression, we examined the impact of reducing 293fectin™ from the recommended range of 40–80 µl (typically 60 µl) per 30 ml culture. We compared the effect of 5, 10, 20 and 30 µl 293fectin™ on IgG expression levels and showed that 293fectin™ can be reduced to 20 µl without any impact on expression levels (data not shown). This represents significant cost savings for the HTP process.

Invitrogen™ recommends that DNA and 293fectin™ are incubated together for 20–30 min before addition to cells. For the parallel transfection of hundreds of samples, this time requirement will interrupt the HTP workflow. To examine the importance of this incubation time, we tested at 10-, 20-, 30-, 45- and 60-min intervals (data not shown). We found all incubation times produced comparable IgG yields, and hence complex addition to cells can occur at anytime between 10 and 60 min.

### IgG expression of InTag reformatted clones

**Figure 5.**
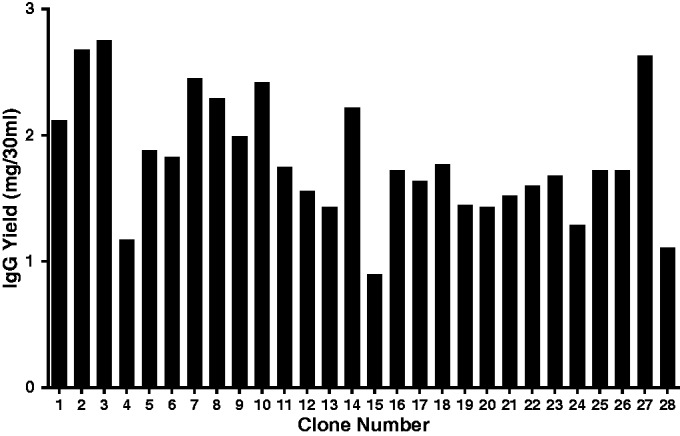
IgG expression levels of InTag reformatted clones. The same 28 unique antibody phage clones described in Figure 3 were expressed as IgGs using our optimized protocol (see online Methods) and antibody levels quantitated by protein A HPLC, where integrated chromatogram elution peaks were compared with reference immunoglobulin standards of known concentration.

To demonstrate the HTP potential of our method, 28 independent phage clones with specificity to the same target antigen were selected from the DYAX ‘Fab-on-phage’ library, reformatted to IgGs using InTag cloning and transiently expressed using our optimized protocols. As shown in Figure 5, all constructs expressed >0.9 mg of IgG per 30 ml culture (30–90 mg/L) and averaged ∼1.4 mg (47 mg/L). These expression levels are significantly higher than those previously reported for the transient expression of IgG reformatted phage clones from the DYAX library (15–20 mg/L) (16). We have expressed >2000 independent phage clones from the DYAX library and have observed that >90% of phage-derived clones are able to be successfully reformatted and expressed at levels sufficient for extensive characterization (>0.5 mg).

Using a HTP and an automated two-step protein purification process based on the Akta Express (GE Healthcare, UK) (see ‘Methods’ section), we routinely achieve antibody recoveries and purity of >80 and 95%, respectively (data not shown). We use a four-module assembly, which can purify up to 2500 proteins per year (20). The addition of arginine in the wash step (21) ensures endotoxin levels are typically < 1.0 EU/mg (data not shown). Low endotoxin levels are particularly important where the functional screening of antibodies uses lipopolysaccharide-sensitive primary cell-based assays.

## DISCUSSION

Owing to the time-consuming and laborious nature of current IgG reformatting methods, most of the initial screening of phage-derived candidates is performed with phage clones or antibody fragments expressed in *E. coli* before selection of a small number of leads for IgG reformatting and functional characterization. This is a major bottleneck for a process that has been highly automated for HTP screening before the important step of testing as IgGs, often the final clinical format. The key limitation with current IgG reformatting methods is the need to plate out and screen individual reformatted candidates due to cloning background in a multi-step cloning process. We describe a novel one-step zero-background cloning method that enables rapid IgG reformatting of antigen-specific phage-derived candidates. Compared with commonly used two-step cloning methods into a single expression vector, InTag cloning can reduce the time required for IgG reformatting by >50% (Supplementary Figure S3). Using optimized transient expression conditions, sufficient amounts of highly pure and endotoxin low IgG material is generated from 30-ml cultures to enable the extensive functional and biophysical evaluation of larger numbers of antibodies, increasing the probability of identifying the most potent antibodies where biological functionality, rather than strict antigen specificity, is required.

Bypassing the need for bacterial expression and characterization of soluble antibody fragments in *E. coli* dramatically decreases the expense and time (∼3 weeks for 50 unique clones) required for the selection of an antibody lead candidate. In addition, we often observe that a significant number of phage clones (up to 30%) fail to express at suitable levels for extensive characterization as soluble Fab fragments using bacterial expression systems (data not shown). The loss of such a large number of clones can significantly compromise lead discovery, particularly in campaigns where antigen specificity and functionality (e.g. specifically blocking enzymic activity) are required, and the most potent antibodies often represent only a small fraction of the overall antigen-specific phage repertoire generated.

We routinely reformat all unique antigen-specific phage clones from a panning campaign into IgGs for further testing (typically <100). While the selection of antigen-specific antibodies is well served with current phage-screening methodologies, where biological function is a key selection requirement, screening in an IgG format is preferable. For example, we often screen phage libraries for antibodies against cytokine receptors that not only bind with high specificity but also block cytokine binding with high potency. In this case, the ability to screen all specific phage binders in parallel using affinity analysis and cellular bioassays in an IgG format (were avidity can potentially compensate for low affinity clones) significantly improves the ability to select biologically active candidates and enables weak inhibitory antibodies to be detected and potentially selected for affinity optimization.

The use of InTag with LIC for ‘non-DYAX’ antibody phage-display libraries requires adaptation of primers to suit the particular phage library being used. Although this may result in the need to generate a number of primers, once obtained they can be called on for all future reformatting needs. While we routinely use In-Fusion® (Clontech) cloning during our IgG reformatting, the InTag adaptor can also be used with other LIC methods. Furthermore, we have shown that traditional ligation can also be used with our method (Figure 2), hence allowing laboratories to integrate InTag-based IgG reformatting into their current protocols without the need to generate new primer sets. As an example, the InTag cloning strategy can be readily applied to the pFab-CMV vector system (15) by converting the SpeI-XbaI fragment into an InTag adaptor. This can be achieved by inserting an *E. coli* selection marker (e.g. CmR) into the SpeI-XbaI fragment at, for example, downstream of either polyadenylation region. As SpeI and XbaI have compatible ends, we recommend the XbaI site be replaced with another unique restriction site to ensure directional cloning.

All steps in InTag IgG reformatting and optimized mammalian expression methods are amenable to automation for HTP screening. Furthermore, the gel filtration step used during protein purification enables clones to be assessed for propensity to form aggregates and provides valuable information as to their future development potential during the initial screening stages (data not shown). The ability to directly screen large numbers of phage-derived antibodies in parallel for binding affinity and functional activity in an IgG format enables lead candidates with optimal characteristics to be rapidly selected. Consequently, we have successfully exploited this method to generate and assess >2000 full-length antibodies targeting a broad range of antigen types resulting in high quality, as assessed by affinity and biological potency, lead reagents and final drug candidates.

## SUPPLEMENTARY DATA

Supplementary Data are available at NAR Online.

Supplementary Data
