# Supplementary material for: One-step zero-background IgG reformatting of phage-displayed antibody fragments enabling rapid and high-throughput lead identification
Source: Nucleic Acids Res. 2013 Nov 16;42(4):e26. doi: 10.1093/nar/gkt1142 (PMC3936716; doi:10.1093/nar/gkt1142)
Supplement: Supplementary Data [file supp_42_4_e26__index.html]

One-step zero-background IgG reformatting of phage-displayed antibody fragments enabling rapid and high-throughput lead identification — One-step zero-background IgG reformatting of phage-displayed antibody fragments enabling rapid and high-throughput lead identification — Supplementary Data 

# One-step zero-background IgG reformatting of phage-displayed antibody fragments enabling rapid and high-throughput lead identification

## Supplementary Data

files

**Files in this Data Supplement:**

- Supplementary Data - pdf file
