# Supplementary material for: One-step zero-background IgG reformatting of phage-displayed antibody fragments enabling rapid and high-throughput lead identification
Source: Nucleic Acids Res. 2013 Nov 16;42(4):e26. doi: 10.1093/nar/gkt1142 (PMC3936716; doi:10.1093/nar/gkt1142)
Supplement: Supplementary Data [file supp_gkt1142_nar-01424-met-g-2013-File006.pdf]

SUPPLEMENTARY INFORMATION

Supplementary Table 1: primers for light chain and VH amplification and reformatting

Primer	Type	Name	Sequence 5′- 3′
P1	Kappa	IFkappa	CTCCACAGGCGTGCACTCTGACATCCAGATGACCCAG
	Lambda	IFayelt	CTCCACAGGCGTGCACTCACAGAGCGAATTGACTCAG
		IFqsalt	CTCCACAGGCGTGCACTCACAGAGCGCTTTGACTCAG
		IFqsvlt	CTCCACAGGCGTGCACTCACAGAGCGTCTTGACTCAG
		IFsyelt	CTCCACAGGCGTGCACTCAAGCTACGAATTGACTCAG
P2	Kappa	IFCKBGHpAr	CACAGTCGAGGCGCGCCTTATTAACACTCTCCCCTGT
	Lambda	IFCLBGHpAr	CACAGTCGAGGCGCGCCTTATTATGAACATTCTGTAG
P3	VH	IFCMVHf	CTCCGAAGTTCAATTGTTAGAGTCTGGTGGCGGCCT
P4		IFhG4NheIR	GGAGCAGGGCGCTAGCGGGAAGACCGATGGGCCTTTG

The primers were adapted from Jostock et al. 2004. As illustrated in Figure 1, Primers P1 and P2 are used to amplify the light chains (VL+CL) and P3 and P4 are used to amplify the VH region from the Fab clones. The 15bp extension required for In-Fusion cloning is either single or double-underlined. They are either complimentary to the vector (double-underlined) or to the InTag adaptor (single-underlined).

Supplementary Table 2: primers sequences for InTag adaptor generation

ID	Name	Sequence 5′- 3′	Product
1	BGHpAf	AAAGGCGCGCCTCGACTGTGCCTTCTAG	BGHpA
2	BGH/CmRr	TTTTACGTTTCTCGTTCAGCCATAGAGCCCACCGCATC	
3	BGH/CmRF	GATGCGGTGGGCTCTATGGCTGAACGAGAAACGTAAAA	CmR
4	CmR/CMVr	GTCAATAATCAATGTCAACTTACGCCCCGCCCTGCCA	
5	CmR/CMVf	TGGCAGGGCGGGGCGTAAGTTGACATTGATTATTGAC	pCMV
6	CMV/SPr	CAGCTCCATCCCATGGTGGCGGCCCTATAGTGAGTCGTA	
7	CMV/SPf	TACGACTCACTATAGGGCCGCCACCATGGGATGGAGCTG	Signal
8	VHspR	GCTGTGCACTCCAGTAGCTG	
9	BGH/ZeoRr	GAGAAAATACCGCATCAGGCCATAGAGCCCACCGCA	BGHpA
10	BGH/ZeoRF	GATGCGGTGGGCTCTATGGGCCTGATGCGGTATTTTC	ZeoR
11	ZeoR/CMVr	TGGCAGGGCGGGGCGTAAGTTGACATTGATTATTGA	
12	ZeoR/CMVf	GCCGAGGAGCAGGACTGACGTTGACATTGATTATTG	pCMV

BGHpA: the bovine growth hormone polyadenylation signal, CmR: chloramphenicol resistance gene, ZeoR: zeocin resistance gene, pCMV: CMV promoter, Signal: antibody secretion signal

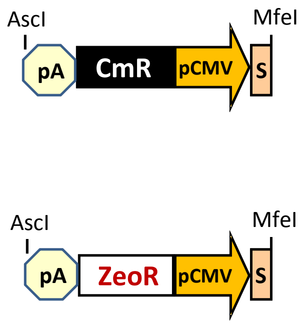

Supplementary Figure 1. Schematic illustration of the InTag adaptors.
pA: the bovine growth hormone polyadenylation signal , CmR: chloramphenicol resistance gene, ZeoR: zeocin resistance gene, pCMV: CMV promoter, S: antibody signal peptide.

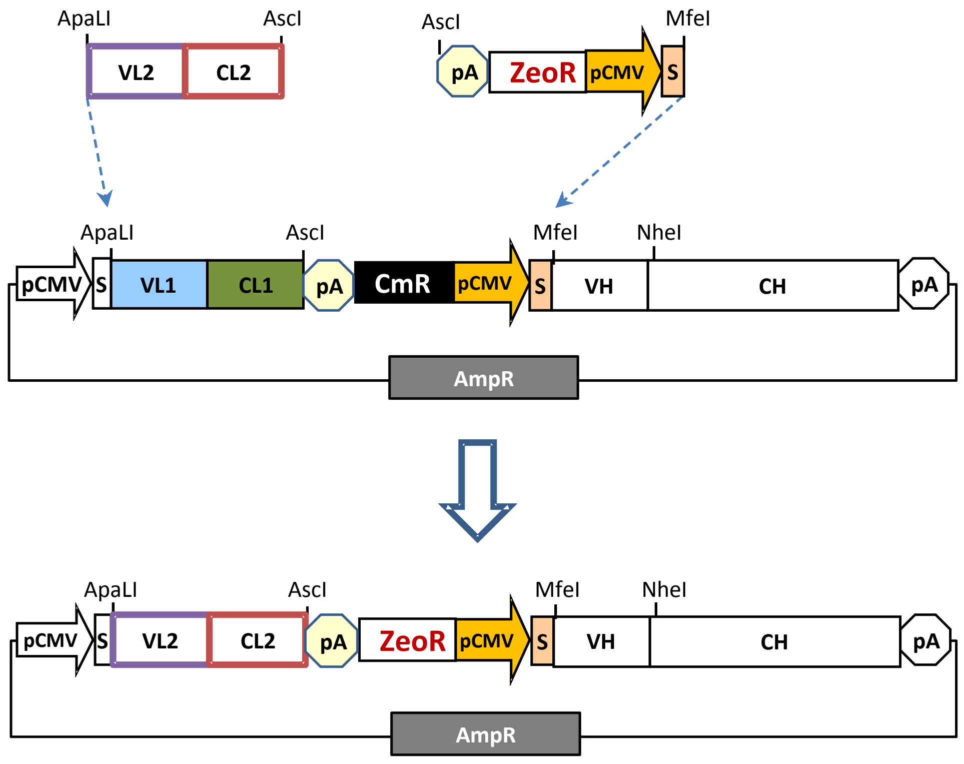

Supplementary Figure 2. Schematic of the use of InTag positive selection for light chain swapping in guided selection strategies
The original light chain (VL1+CL1) and the CmR InTag adaptor were removed from the IgG expression vector by ApaLI and MfeI digestion. A new light chain (VL2+CL2) was cloned into the expression vector along with the ZeoR InTag adaptor at the ApaLI and MfeI sites. The recombinant clones containing the new light chain were selected with Zeocin. The VH region can be similarly swapped using the AscI and NheI cloning sites.

pA: the bovine growth hormone polyadenylation signal, CmR: chloramphenicol resistance gene, ZeoR: zeocin resistance gene, pCMV: CMV promoter, S: antibody secretion signal.VL1: variable light region of antibody 1, VL2: variable light region of antibody 2, CL1: light constant region of antibody 1, CL2: light constant region of antibody 2.

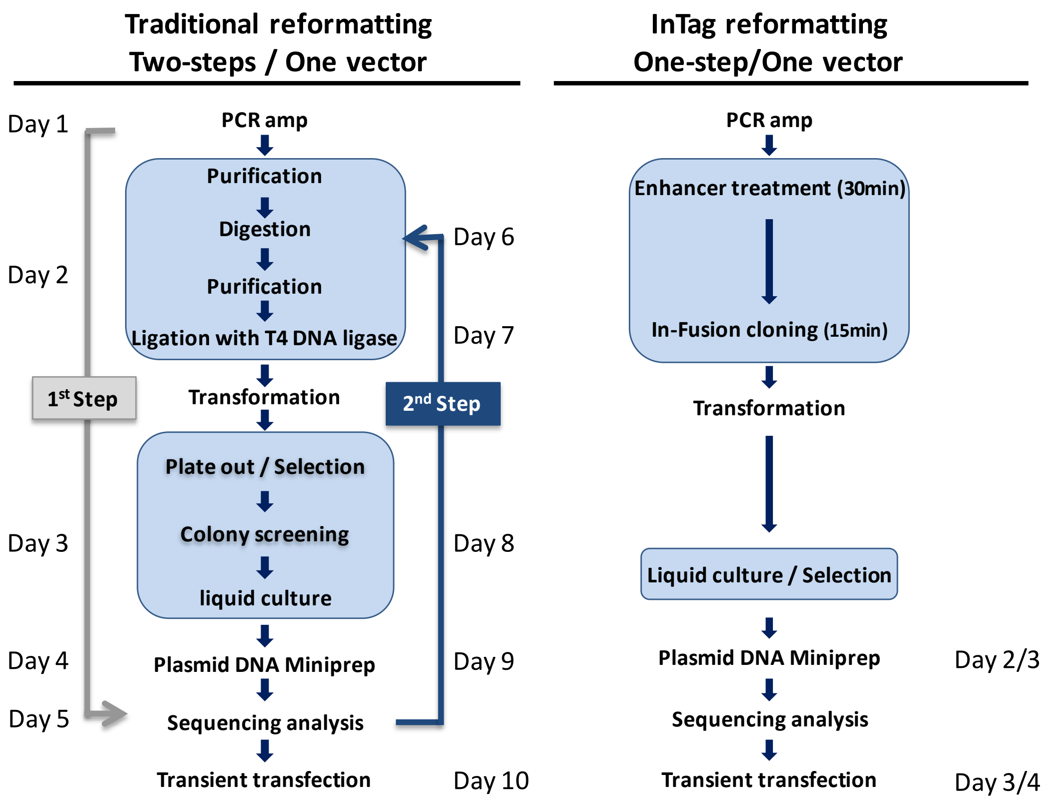

Supplementary Figure 3. Comparison of workflows
Comparison of InTag IgG reformatting with traditional two-step cloning using a single expression vector. InTag IgG reformatting method dramatically simplifies the cloning procedure and increases the throughput.
